# Adaptation and early implementation of the PREdiction model for gene mutations (PREMM_5_™) for lynch syndrome risk assessment in a diverse population

**DOI:** 10.1007/s10689-021-00243-3

**Published:** 2021-03-23

**Authors:** Kathleen F. Mittendorf, Chinedu Ukaegbu, Marian J. Gilmore, Nangel M. Lindberg, Tia L. Kauffman, Donna J. Eubanks, Elizabeth Shuster, Jake Allen, Carmit McMullen, Heather Spencer Feigelson, Katherine P. Anderson, Michael C. Leo, Jessica Ezzell Hunter, Sonia Okuyama Sasaki, Jamilyn M. Zepp, Sapna Syngal, Benjamin S. Wilfond, Katrina A. B. Goddard

**Affiliations:** 1 Department of Translational and Applied Genomics, Center for Health Research, Kaiser Permanente Northwest, Portland, OR USA; 2 Dana Farber Cancer Institute, Boston, MA USA; 3 Harvard Medical School, Boston, MA USA; 4 Center for Health Research, Kaiser Permanente Northwest, Portland, OR USA; 5 Institute for Health Research, Kaiser Permanente, Denver, CO USA; 6 Denver Health and Hospital Authority, Denver, CO USA; 7 Brigham and Women’s Hospital, Boston, MA USA; 8 Treuman Katz Center for Pediatric Bioethics, Seattle Children’s Research Institute and Hospital, Seattle, WA USA; 9 Department of Pediatrics, University of Washington School of Medicine, Seattle, WA USA

**Keywords:** Family history, Hereditary cancer, Risk assessment, Lynch syndrome, Underserved

## Abstract

Lynch syndrome (LS) is the most common inherited cause of colorectal and endometrial cancers. Identifying individuals at risk for LS without personal cancer history requires detailed collection and assessment of family health history. However, barriers exist to family health history collection, especially in historically underserved populations. To improve LS risk assessment in historically underserved populations, we adapted the provider-facing PREdiction Model for gene Mutations (PREMM_5_™ model), a validated LS risk assessment model, into a patient-facing electronic application through an iterative development process involving expert and patient stakeholders. We report on preliminary findings based on the first 500 individuals exposed to the adapted application in a primary care population enriched for low-literacy and low-resource patients. Major adaptations to the PREMM_5_™ provider module included reduction in reading level, addition of interactive literacy aids, incorporation of family history assessment for both maternal and paternal sides of the family, and inclusion of questions about individual relatives or small groups of relatives to reduce cognitive burden. In the first 500 individuals, 90% completed the PREMM_5_™ independently; of those, 94% did so in 5 min or less (ranged from 0.2 to 48.8 min). The patient-facing application was able to accurately classify 84% of patients as having clinically significant or not clinically significant LS risk. Our preliminary results suggest that in this diverse study population, most participants were able to rapidly, accurately, and independently complete an interactive application collecting family health history assessment that accurately assessed for Lynch syndrome risk.

**Supplementary Information:**

The online version contains supplementary material available at 10.1007/s10689-021-00243-3.

## Introduction

Lynch syndrome (LS) is the most common hereditary cause of colorectal cancer (CRC), accounting for about 2–3% of all CRC [1–5]. Individuals with LS have increased lifetime risk of colorectal and endometrial cancers and increased risk for other malignancies such as stomach, small bowel, pancreas, ovarian, brain and urinary tract cancers [6]. Despite this increased risk, early identification of patients with LS allows for regular surveillance and risk-reduction procedures that can lead to decreased cancer incidence and mortality [6, 7]. While increasing awareness of hereditary cancer syndromes has led to more genetic predisposition testing for hereditary cancers, much of the increase in demand appears to be driven by testing for the more well-known hereditary breast and ovarian cancer syndrome (*BRCA1* and *BRCA2*), while referral and testing for Lynch syndrome has remained relatively constant [8, 9]. Currently, the vast majority of individuals who carry LS mutations (*MLH1*, *MSH2*, *MSH6*, *PMS2*, and *EPCAM*) remain undiagnosed [9, 10]. As such, tools that facilitate LS identification are needed.

While systematic efforts to improve LS detection through universal tumor screening of patients with LS cancers exist, diagnosis of LS in the cancer-free population relies on a multi-step process initiated by the identification of a close relative with LS or a concerning family history. Insufficient family health history assessment and provider discomfort with obtaining family history negatively impacts the evaluation of hereditary cancer risk [11–13]. As a result, many individuals with LS mutations remain unidentified among the cancer-free population [11–13]. Family health history information about cancer has often been limited to first-degree relatives and may miss key details such as type of cancer and/or age at diagnosis [14–16]. Even when family health history is collected accurately and completely, appropriate genetics services referrals still may not take place [17].

In addition to barriers to obtaining accurate family health history, there are disparities in provision of referral for hereditary cancer genetic evaluation in historically underserved populations, leaving these individuals even less likely to have an appropriate risk assessment [18–22]. Emerging research shows that health information technology applications that utilize patient-entered family health history information can improve identification of individuals at-risk for hereditary cancers or other genetic conditions [23, 24]. Yet despite the promise of this research, significant barriers remain for equitable access to precision health care and personalized medicine, especially for historically underserved populations [19, 20, 22, 25–28]

To address the important issue of LS identification in cancer-free individuals, especially from historically underserved groups, we are conducting the Cancer Health Assessments Reaching Many (CHARM, NCT03426878) study as part of the Clinical Sequencing Evidence-Generating Research consortium [29]. This study is working to improve family health history-based access to genetic services for hereditary cancer syndromes. We used an iterative process to adapt a validated provider-facing risk assessment algorithm for LS—the PREdiction Model for gene Mutations-5 (or PREMM_5_™ model) [30], into a patient-facing electronic application. This tool was originally designed to help healthcare providers obtain a streamlined family history to identify individuals who should undergo genetic evaluation for LS. The adaptation process in the CHARM study included feedback from Patient Advisory Councils (PACs) and tool refinement by a large team of multidisciplinary healthcare delivery experts. We have now implemented this application in a primary care population enriched with individuals from medically underserved communities, and here we describe (1) the process and outcomes of the adaptation of the PREMM_5_™ model into a patient-facing application and (2) the initial implementation of the PREMM_5_™ patient application in two large healthcare settings reaching racially and ethnically diverse and low-socioeconomic status populations. If successful, the PREMM_5_™ patient application could be incorporated into primary care, reducing the barriers to successful identification of individuals at risk for LS.

## Methods

### Study overview

CHARM is testing a novel cancer genetics care delivery paradigm in predominantly healthy populations ages 18–49 years in the primary care setting. As part of the study, individuals are assessed for eligibility for genetic testing using validated hereditary cancer risk assessment algorithms.

### Study setting

The setting included two healthcare delivery systems, Kaiser Permanente Northwest (KPNW) and Denver Health (DH), which have distinct clinical populations. KPNW is a vertically integrated system serving approximately 607,000 members in Northwest Oregon and Southwest Washington. We recruited patients scheduled to see providers located at two KPNW clinics with a higher proportion of patients who are racially or ethnically diverse and/or live in census tracts with > 20% of residents below the federal poverty level or have > 20% of residents with less than a high school education, henceforth referred to under the umbrella term “underserved populations.” DH is an integrated safety-net health system with nine Federally Qualified Health Centers based in Denver, Colorado. DH serves approximately 160,000 patients in Denver County, 69% of whom are racial and ethnic minorities, 81% of whom are publicly insured or uninsured, and 77% of whom live at or below 200% of the federal poverty level.

While we sought to recruit a disproportionately higher number of medically underserved populations, all English- or Spanish-speaking patients ages 18–49 years from both KPNW and DH health systems were eligible to take the risk assessment. The KPNW Institutional Review Board (IRB) approved this study and all collaborating IRBs ceded to KPNW except Dana Farber Cancer Institute and Columbia University, who approved the study separately.

### Adapting the PREMM_5_™ provider web-based module into a patient-facing electronic application

#### Patient advisory committees (PAC)

DH and KPNW healthcare providers identified patients potentially interested in participating in CHARM’s Patient Advisory Committees (PAC). We recruited seven DH patients whose primary language was English to provide input on the English version of the PREMM_5_™ patient application (English-language PAC). Five of the seven English-language PAC members were from racial or ethnic minority groups. For the Spanish-language PAC, we recruited a group of 10 native Spanish speakers: three from DH and seven from a single KPNW clinic that primarily serves Spanish-speaking patients.

#### Iterative adaptation

The adaptation team included a Spanish-language and health literacy expert, two original developers of the PREMM_5_™ model, multiple healthcare delivery experts, a software engineer, and a user interface/experience design expert. The adaptation team developed the English patient-facing PREMM_5_™ application using the provider-facing PREMM_5_™ web-based module and the patient-facing PREMM_1,2,6_ electronic application implemented in a community practice setting [30, 31]. Initial adjustments were made for literacy level and health literacy by the health literacy expert. Following this input, the software design team created wireframe models of skip logic and graphical aids.

**Fig. 1:**
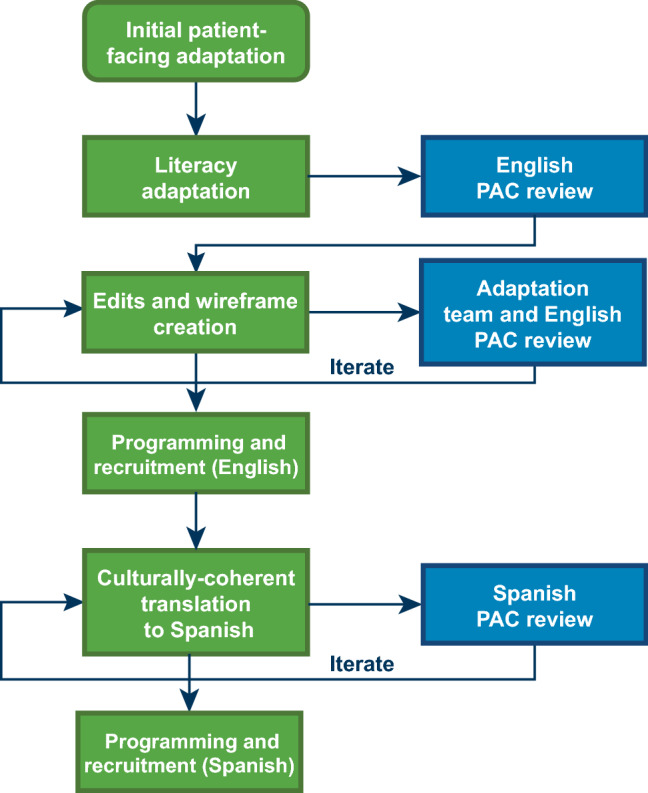
Depiction of iterative adaptation and implementation of the patient-facing version of PREMM_5_™. Following initial adaptation of the provider-facing tool, a health literacy expert performed literacy adaptation. The English-speaking PAC provided feedback on these materials, and this feedback was incorporated into wireframes that were iteratively reviewed by the study team and English-speaking PAC. The Spanish-speaking PAC provided iterative feedback on crucial aspects of the culturally-coherent translation

The English adaptation team, English-language PAC, and CHARM study team iteratively reviewed these adapted materials (Fig. 1) [32]. During three PAC meetings and individual cognitive interviews, English-language PAC members provided feedback regarding the explanation of the purpose of the risk assessment, adapted question and results wording, layout of the tool, and made recommendations for where literacy aids would be helpful. The Spanish-language PAC provided input on Spanish translation wording and additional needs of the Spanish-speaking population in three rounds of individual cognitive interviews (Fig. 1). A separate manuscript will describe the PAC process and contributions to the CHARM study as a whole.

**Fig. 2:**
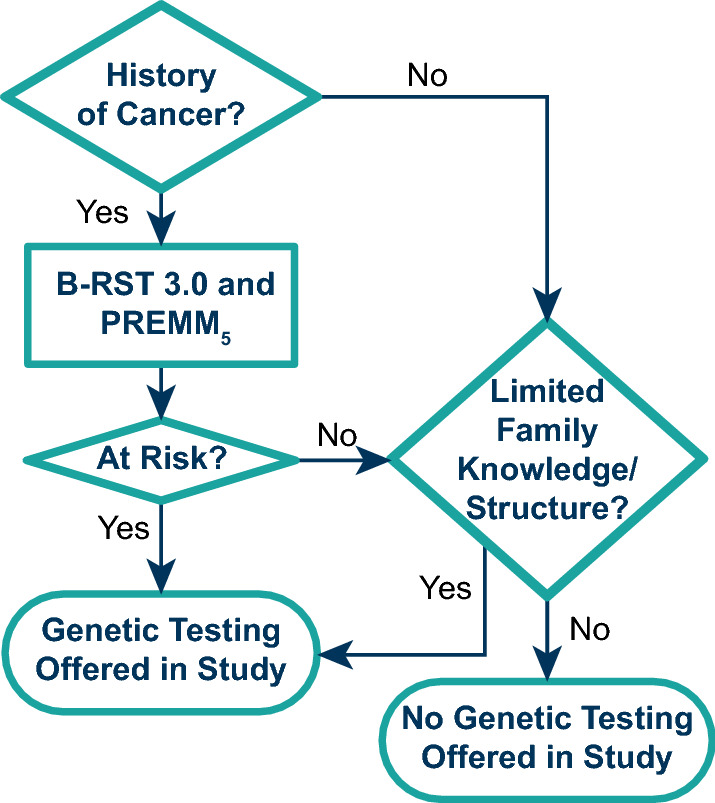
Patient pathing through study eligibility criteria assessment, including the PREMM_5_™ tool. Eligibility by PREMM_5_™ corresponds to a risk score of ≥ 2.5%; eligibility by B-RST™ 3.0 corresponds to a moderate or high risk score; eligibility by additional questions implies that the patient had limited family knowledge (unknown family history) or limited family structure (< 2 female members living beyond age 45 on either side)

A patient-facing integrated version of the Breast Cancer Genetics Referral Screening Tool (B-RST™ 3.0) for individuals of limited literacy was developed in a parallel process that will be reported elsewhere [33–35]. In the CHARM eligibility assessment, the PREMM_5_™ and B-RST™ 3.0 applications were combined into a single sequence with a novel third module which evaluated limited family history (Fig. 2). Importantly, this sequence began with a set of questions inquiring about the patient’s personal and/or family history of cancer; responses to these initial questions determined to which specific risk assessment questions individuals were exposed. For individuals with a personal and/or family history of cancer, they are exposed to the B-RST™ 3.0 and PREMM_5_™ applications in a randomized fashion. B-RST™ 3.0 consists of questions regarding the personal and family (first- and second-degree relatives’) history of breast cancer and ovarian cancer, as well as Ashkenazi Jewish ancestry. PREMM_5_™ consists of questions regarding personal and family (first- and second-degree relatives’) history of colon cancer, endometrial cancer, and the following LS-associated cancers: ovarian, stomach, small intestine, urinary tract/bladder/kidney, bile duct, brain, and pancreatic, as well as sebaceous gland skin tumors. The novel limited family history module asks about patient history of adoption, patient knowledge of cancer on the paternal/maternal sides, and the number of female relatives living beyond the age of 45 on each side. This module also screens for past history of genetic testing for LS or hereditary breast and ovarian cancer.

### Identification and enrollment

#### Participant identification and mailings

At KPNW, we used electronic medical records to identify patients with upcoming appointments at participating clinic sites and recruited them via email and text message and set up in-clinic recruitment booths at two KPNW clinic sites, beginning in August 2018. At DH, patients who were identified as at potentially higher risk via the electronic medical record were recruited via postcard, follow up phone calls, and provider referral; patients at potentially higher risk were defined as (1) receiving cancer screening outside of general population screening guidelines, (2) having a hereditary breast and ovarian cancer syndrome- or LS-related cancer diagnosis before age 50, (3) documentation of family history of cancers related to either syndrome, or (4) receiving a referral for cancer-related genetics services but no documentation of genetic testing. This method was chosen as the starting recruitment approach at DH to facilitate patient receipt of genetic testing in this low-resource setting. Recruitment at DH began in October 2018. Preliminary results from the first 500 individuals encountering the PREMM_5_™-specific portion of the CHARM eligibility assessment and the first 124 individuals receiving genetic test results through the study are reported herein. Recruitment in Spanish began in February 2019, due to the phased implementation of translation and Spanish-language PAC review, as well as study resource requirements for programming the Spanish-language electronic tools.

#### Eligibility assessment and consent process

Interested patients could access CHARM eligibility assessment and study consent via the study webpage on their personal electronic device or, if assessment was completed at the clinic, on a clinic-provided tablet. Optional bilingual (English/Spanish) support for individuals completing the risk assessment was available in-person in the clinic, or over the phone. Following consent for the risk assessment, the application presented initial questions about personal or family cancer history. If the individual reported either personal or family history of cancer, the PREMM_5_™ assessment and the B-RST™ 3.0 assessment were presented in random order (Fig. 2). If individuals reported no personal or family history to the initial questions, they were only presented with additional questions assessing family structure and family history knowledge. A negative screen on both the PREMM_5_™ assessment and the B-RST™ 3.0 assessment also resulted in assessment of family structure and knowledge.

#### Study enrollment and multi-step consent process for genetic testing

The PREMM_5_™ application provided immediate feedback about LS risk and a report about indications for genetic testing in the case of a clinically significant PREMM_5_™ risk score (≥ 2.5%). Participants electing to receive genetic testing through the study completed a baseline survey and received genetic testing results during a post-test telephone genetic counseling session. Individuals who were eligible for genetic testing but declined to enroll in the study received an optional decliner survey. The baseline and decliner surveys included questions on years of formal education and English fluency, and the baseline survey assessed numeracy using a validated subjective numeracy measure, the three-item subjective numeracy scale (SNS-3). Responses have a summed range of 3–18, with higher scores representing higher numeracy [36]. We report numeracy as an important measure, because the PREMM_5_™ application requires patients to add relatives together (e.g., number of brothers, sisters, and children with colorectal cancer) and to choose the youngest affected relative among that subgroup.

### Collection of quality metrics

The total population included in our report was 517 individuals. These included the first 500 participants exposed to PREMM_5_™-specific questions with complete data, and 17 additional individuals included in the validity analysis.

#### Time to completion

Our automated tracking system recorded the individual’s language of choice, time of PREMM_5_™ answer input, answers to specific questions, and the actual PREMM_5_™ scores for both sides of the family. For the first 500 individuals exposed to PREMM_5_™-specific questions and who completed the entire CHARM risk assessment, time to complete the PREMM_5_™ portion of the application was computed as the time participants spent on PREMM_5_™-specific questions. In the same population of the first 500 individuals exposed to PREMM_5_™-specific questions, the number of incomplete CHARM risk assessment encounters is separately reported, along with total time spent on the entirety of the CHARM risk assessment and the PREMM_5_™ portion, though participants may have terminated interaction during completion of the PREMM_5_™ portion.

#### Comparison to genetic counselor-collected family history

In accordance with the adaptation of the Centers for Disease Control ACCE framework adaptation recommended for evaluating family history tools, we assessed the preliminary analytic validity (how well it measures the family history that it intends to collect in comparison to a gold standard family health history collection) of the PREMM_5_™ patient-facing application using data gathered by genetic counselors as the comparator [37–41]. Genetic counselors recorded structured family health history data in a REDCap database from pedigrees constructed during the post-test genetic counseling session, and also independently computed and recorded a participant PREMM_5_™ score using the PREMM_5_™ provider-facing tool on the basis of the genetic counselor-determined affected side of the family.

We compared overall score computed by the patient-facing application to overall score determined by the genetic counselors, using the age at the time of results disclosure. We included all patients with available genetic counseling data (N = 124 at the time of analysis) in the analysis, including (1) those who were not exposed to PREMM_5_™-specific questions and/or did not have a score based on patient-input (i.e. they reported having no personal or family history of LS cancers on the patient-facing application) but who did have a genetic counselor-computed score (i.e. they subsequently reported LS family history of LS cancers to the genetic counselor) and (2) those who had a score based on patient-input but for whom genetic counselors determined that there was no family history of LS cancers. Assuming a minimum acceptable value (H0) of a CCC of 0.80 [42] and a one-tailed alpha value of 0.05, a sample size of 124 provides at least 83% power if the true (H1) CCC is 0.87 or higher. We conducted the power analysis using PASS 15 (PASS 15 Power Analysis and Sample Size Software (2017). NCSS, LLC. Kaysville, Utah, USA, ncss.com/software/pass.) In group (1), we included patients who interacted with the shared questions about overall personal or family history of cancer and were pathed directly to the limited family history questions (were not exposed to PREMM_5_™-specific questions), as they could represent false negatives. The PREMM_5_™ risk score was synthetically set to 0 in the case of no reported LS personal or family history in order to include both of these subsets of patients in the analysis and to differentiate the extent of the difference in the participant self-report and genetic counselor-computed risk scores. We evaluated the agreement between the PREMM_5_™ and genetic counselor calculation using Lin’s concordance correlation coefficient [43].

Scores on the PREMM_5_™ patient application and by genetic counselor calculation that were both ≥ 2.5% (the clinically significant risk score) were considered true positives. True negatives were considered non-clinically significant scores on both the patient application and by genetic counselor calculation. False positives were considered clinically significant scores on the patient application but not by genetic counselor calculation, and false negatives were non-clinically significant scores on the patient application but clinically significant scores by the genetic counselor calculation. An a priori definition of ≥ 5% absolute value difference between the genetic counselor-computed score and the score based on participant input was considered highly discrepant.

To determine whether sociodemographic characteristics were predictive of whether or not someone was one of the 124 individuals in the mutually exclusive subset from the 500 individuals included in the analyses of time spent on PREMM_5_™, we used logistic regression with the exception of language which was evaluated via a Fisher’s exact test. We performed separate analyses for each sociodemographic characteristic on the outcome of subset membership. All inferential tests were evaluated a two-tailed alpha level of 0.05, but focused mainly on the magnitude based on the odds ratio.

## Results

### Iterative adaptation of the provider web-based PREMM_5_™ module

The adaptation team advocated three major changes to the provider web-based PREMM_5_™ module: (1) to not rely upon the patient to determine the affected side of the family (i.e., the side of the family with LS cancers); (2) to inquire about relatives one at a time or in small groups, rather than asking the patient about first and second-degree relatives, to reduce the demand for high numeracy; (3) to embed literacy aids [31].

**Fig. 3:**
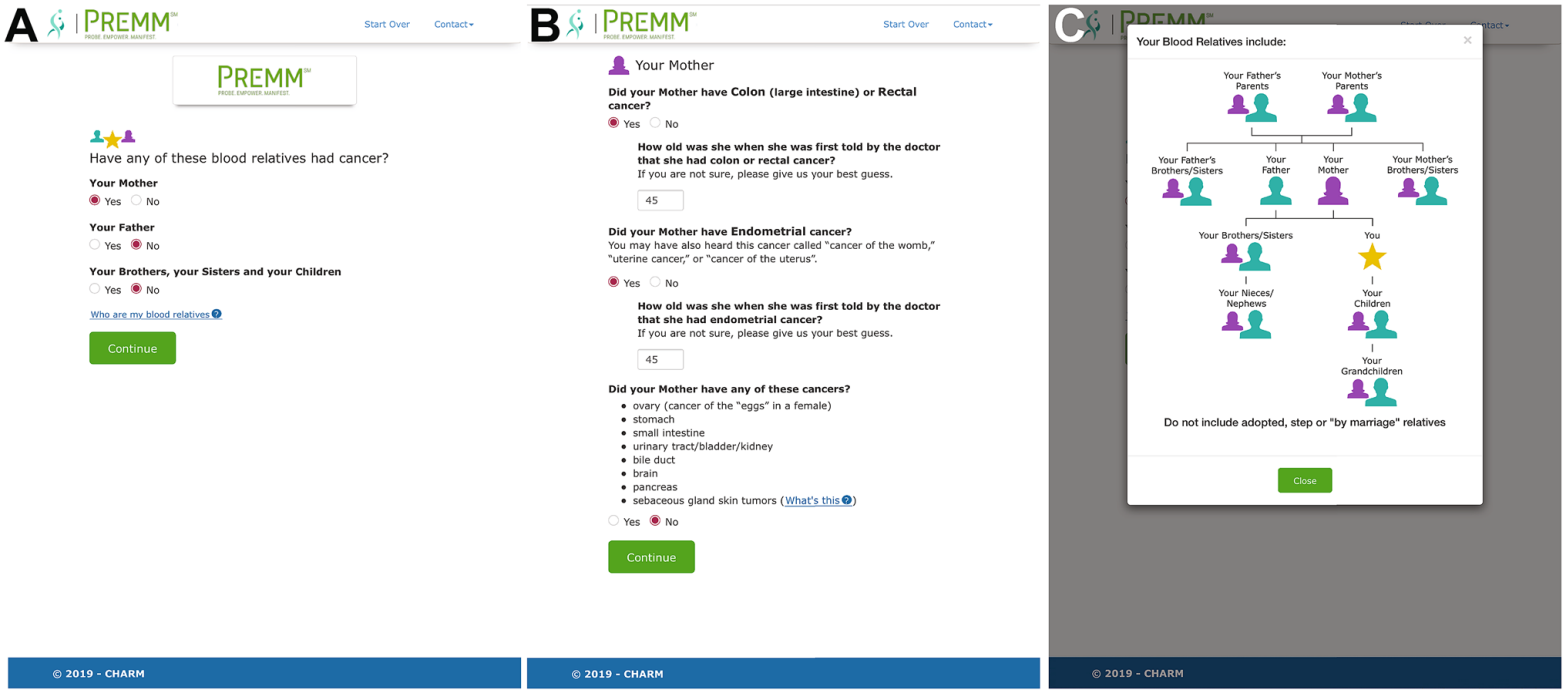
Sample images from the patient-facing PREMM_5_™ application. **a** Sample screen inquiring about cancer history in individual relatives or small groups of relatives. **b** Sample screen inquiring about cancer history in the mother, which appears if the participant selects that the mother had cancer. **c** Sample literacy aid pop-up window depicting the family tree graphic, which appears if the patient selects the modal link titled “Who are my blood relatives?” on the screen in A. The pop-up literacy aid for sebaceous gland skin tumors that appears when the patient selects the modal link in B is depicted in Supplementary Fig. 2a

In order to successfully implement adaptations (1) and (2) in a patient-friendly manner, user-experience design experts developed skip logic and a layout that presented only a few questions per screen and employed responsive design elements, to allow for ease of implementation on a variety of patient devices, ranging from desktop to handheld smartphones. Initial questions asked patients if they had a personal or family history of cancer, and in the event of a family history of cancer, the patients answered a second set of questions to narrow down the family members affected by cancer on both sides of the family (Fig. 3a; Supplementary Fig. 1a). For individuals identified as having a personal or family history of cancer, the specific LS-related cancer questions were then presented (Fig. 3b; Supplementary Fig. 1b). To implement the final recommendation (3), the study team embedded hyperlinks to pop-up windows within the interactive application (Fig. 3c; Supplementary Fig. 1c; Supplementary Figure 2).

The PAC provided several suggestions about wording and literacy aids, often providing new phrases to describe biological concepts such as using the phrase “cancer in your family tree” to replace “hereditary cancer” or “inherited cancer” in the explanation and consent for the risk assessment. The PAC also suggested that we clarify “sebaceous gland skin tumors” to minimize it being confused with other more common types of skin cancer. In response, the study team embedded a pop-up literacy aid to describe that cancer (Supplementary Figure 2). The PAC suggested using a family tree graphic (Fig. 3c; Spanish translation in Supplementary Figure IC) to explain biological relatives and recommended the addition of the term “blood relatives.” Finally, the PAC suggested that an explanation of LS be added to the results page (Supplementary Figure 3) and that the study recommend genetic testing if the participant had a clinically-significant risk. However, due to IRB restrictions, the study team was unable to incorporate this suggestion. The Spanish-language PAC provided feedback and recommended, for example, that the tool provide examples of biological relatives and include the clarification that first cousins or in-laws should not be considered as biological relatives.

To support the changes to the tool described above, the PREMM_5_™ output for the patient application algorithm was adapted to compute both a maternal and paternal PREMM_5_™ score, to assign the affected side of the family computationally, and to report the participant PREMM_5_™ result on the basis of the assigned affected side [30]. A comparison of the provider web-based PREMM_5_™ module and the patient-facing application developed in CHARM is presented in Table 1.

**Table 1:** Comparison of key features in the PREMM_5_™ provider module and the PREMM_5_™ patient-facing application

Feature	PREMM_5_™ provider module			PREMM_5_™ patient application
Question Presentation	One scrolling screen			Few questions per screen
Affected side of family	Provider selects the affected side of the family			Removed requirement to select an affected side of the family
Number of questions	9–18, depending on provider answers			2–46, depending on patient answers
Skip logic	Limited			Extensively utilized to streamline the user’s experience
Family health history assessment	Relatives assessed in medically meaningful groups (for e.g., all FDRs)			Relatives assessed in familially meaningful groups/individuals (for e.g., brothers, sisters and children)
Clarifications/Literacy aids	On-screen definitions			Embedded definitions and infographics; further explanation of medical terms (e.g., sebaceous gland skin tumors)
Language	Assumes provider literacy level; English only			Reduced literacy language (removed terms such as “first-degree relatives”); English and Spanish
Similarities	Responsive design (can be used on all devices)			Responsive design (can be used on all devices)

### Participation and demographics

We assessed the first 500 individuals to encounter the adapted PREMM_5_™-specific questions in English (n = 486) or Spanish (n = 14) and evaluated the accuracy of family health history information collected for all study participants that had completed their genetic counseling result disclosure visit at the time of analysis (n = 124) with genetic counselor-collected family health history data. Demographic characteristics are summarized in Table 2 and Supplementary Table 1, and the flow of the total population of participants through the risk assessment is presented in Supplementary Fig. 4.

**Table 2:** Demographic information for the first 500 participants exposed to the patient-facing PREMM_5_™-specific questions in the patient-facing application

Variable	N (%)*
Sex assigned at birth	
Male	95 (19)
Female	405 (81)
Site	
DH	115
KP	385
Application language selection	
English	486 (97)
Spanish	14 (3)
Education^†^	
Less than high school	7 (2)
Some high school	10 (3)
High school graduate	34 (12
Some post-high school training	70 (24)
Associate or vocational degree	30 (10)
Bachelor's degree	55 (18)
Graduate or professional degree	40 (14)
Not provided	47 (16)
English fluency^†‡^	
Native English-speaker	199 (40)
Perfectly well/Very well	24 (5)
Well/Not well	10 (2)
No English	4 (1)
Not provided	263 (53)

Variable	Average	SD	Min	Max
Age (years)	35.3	8.3	18	49
SNS-3 score^§^	12.9	4.0	3	18

^*^All percentage values given out of the population of 500 individuals exposed to PREMM_5_™-specific questions

^†^Percentages are given out of the number of individuals completing the baseline (n = 275) and decliner (n = 18) surveys, which were available only to participants with a genetic testing-eligible risk assessment outcome

^‡^Individuals were considered native English speakers if they indicated they did not speak another language besides English on the baseline survey. English fluency categories were exposed to individuals taking the survey in Spanish and those who indicated they spoke another language besides English

^§^Assessed only for individuals completing this portion of the baseline survey, which was available only to participants with a genetic testing-eligible risk assessment outcome

Most participants (93%; n = 466) interacted with the risk assessment outside of the clinic, while a small proportion (7%, n = 34) did so in clinic; however, 12% (14/115) of DH participants completed the assessment in clinic while 4% (20/385) of KP participants did so, likely reflecting the difference in recruitment approaches at the two sites. A minority (3%, n = 14) selected Spanish-language as their language of choice, likely reflecting the delayed implementation of the Spanish-language option for the risk assessment. Of the individuals using the Spanish version, 79% (11/14) interacted with the risk assessment in clinic compared to 5% (23/486) of individuals using the English version.

Ninety five percent (n = 476) of individuals who encountered the PREMM_5_™-specific questions went on to complete the entire CHARM risk assessment, and of those individuals who completed the assessment, 95% (n = 452) did so without assistance. Of the assisted individuals (N = 24), 29% (N = 7) were documented to have utilized study staff assistance over the phone and 71% (N = 17) utilized study staff assistance in person. Of the 24 assisted individuals, nearly all (96%; n = 23) met the study recruitment definition of an underserved population and a significant proportion (46%, n = 11) were individuals who took the risk assessment in Spanish; 79% of the individuals who took the risk assessment in Spanish utilized assistance compared to only 3% of individuals using the English version. Of those individuals exposed to PREMM_5_™-specific questions who did not complete the full CHARM risk assessment, 29% (n = 7) were from a historically underserved population, as defined by the study, compared to 58% (n = 276) from a historically underserved population among those who completed the risk assessment.

### PREMM_5_™ patient application completion

We examined the time participants spent on PREMM_5_™-specific questions. Ninety four percent (n = 423) of individuals who independently completed the CHARM risk assessment spent 5 min or less on PREMM_5_™-specific questions. The average time spent to complete the PREMM_5_™ application was 2.3 min (SD = 3.6; ranged from 0.2 to 48.8 min). Only two participants took longer than 30 min to complete the PREMM_5_™-specific questions. On average, participants were exposed to 13 (SD = 3; ranged from 2 to 29) PREMM_5_™-specific questions.

It is possible that participants who did not complete the CHARM risk assessment may have spent a longer time on the risk assessment and/or on the PREMM_5_™ portion. We evaluated the overall time spent on the CHARM risk assessment, as well as the time on PREMM_5_™ alone, for all sessions where the CHARM risk assessment was not completed. Considering all incomplete CHARM risk assessments with exposure to PREMM_5_™-specific questions, 82% of sessions had a CHARM risk assessment exposure duration lasting five or fewer minutes, while 89% of the PREMM_5_™-specific exposure durations lasted five or fewer minutes.

### Preliminary comparison of patient-reported family history to family history collected by genetic counselors

At the time of analysis, 124 participants had completed their post-test genetic counseling session with a genetic counselor through the CHARM study, 108 of whom were exposed to PREMM_5_™-specific questions and 16 of whom pathed directly to the limited family history module due to lack of reported personal or family history of cancer. The associations of sociodemographic characteristics with whether or not an individual was included only in the time and incompletion analyses are presented in Supplementary Table 1; that application language is predictive of inclusion in the validity analysis was expected based on lack of availability of the Spanish language tool at study start. Among these 124 participants, 20 participants (16%) were mis-categorized from participant-generated PREMM_5_™ risk scores in comparison to genetic counselor-computed PREMM_5_™ risk scores, and were all deemed false positives, including 10 who were determined to have no LS-related cancer history. Participant-generated PREMM_5_™ risk scores identified all participants who had clinically significant genetic counselor-computed PREMM_5_™ scores, as such there were no false negatives in the subset of participants examined. Notably, 25% (31) of the participants examined had a clinically significant PREMM_5_™ risk score on the patient-facing application, with or without qualifying scores on the other CHARM risk assessment algorithms, while 75% (93) qualified for genetic testing on the basis of the other risk algorithms only. Four participants did not input personal LS cancer history but were determined to have personal LS cancer history by the genetic counselor but achieved clinically insignificant PREMM_5_™ scores.

**Fig. 4:**
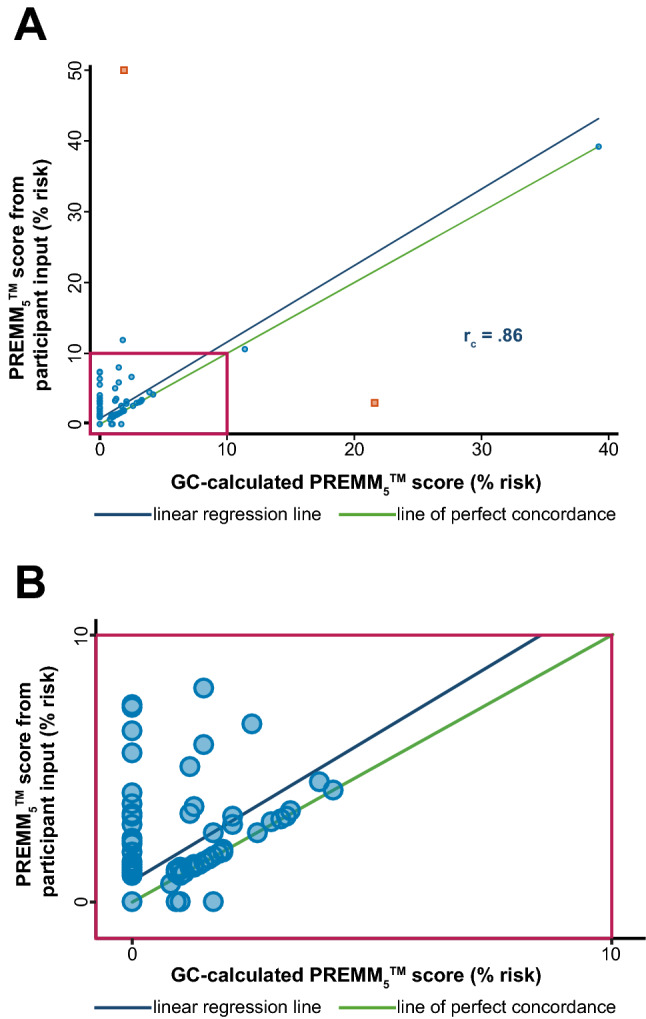
Patient and provider differences in PREMM_5_™ risk scores. **a** Scatter plot of PREMM_5_™ scores derived from participant input and from genetic counselor-collected pedigrees. r_c_ = concordance correlation coefficient after removal two of outliers (orange, square points). Concordance correlation coefficient with the two extreme outliers included was 0.51. Magenta box = inset. **b** Magnified view of magenta inset in Fig. 4a, demonstrating the trend toward participant overreport on the patient-facing tool when there was disagreement between measures, as well as the excellent agreement in the two measures for some participants (points on the line of perfect concordance)

Comparing the risk score difference between genetic counselor-computed PREMM_5_™ risk scores and those derived from participant input, participant completed family health history is more likely to overreport family history compared to the genetic counselor-collected family history. In the original assessment of agreement including all 124 data points, the concordance correlation coefficient was 0.51, 95% CI [0.40, 0.63]. However, there were two extreme multivariate outliers (absolute value difference in scores ≥ 15%). Upon removing those outliers, the concordance correlation coefficient was 0.86, 95% CI [0.82, 0.91] (Fig. 4), indicating that these were extremely influential observations and thus the inclusion of these points mask the true level of agreement observed for > 98% of the cases. Eight participants (6.5%) had patient-derived PREMM_5_™ scores with a score difference of 5% or greater compared to their genetic counselor-computed score, meeting our a priori score difference cut-off. The genetic counselor-computed PREMM_5_™ scores were lower than patient-derived PREMM_5_™ scores in seven of the eight participants, and all eight discrepant scores were due to varying magnitudes of misreported family health history in first- and second-degree relatives. Similarly, discernible patterns were not observable in the 20 individuals with false positive risk scores, and more data will be needed to evaluate any trends that could be addressed by risk assessment modifications. Future work will include qualitative interviews with study participants to evaluate their perception of the PREMM_5_™ assessment, including patient perceptions of reasons for misreport, and a detailed assessment of patterns found in relative type(s) or type(s) of cancer leading to patient misreport.

## Discussion

With the goal of mitigating barriers to LS risk recognition and LS-related genetics services referral, we developed a patient-facing PREMM_5_™ application. This application is designed to facilitate self-reported cancer family health history by patients at lower levels of health literacy in the primary care setting. Our preliminary results suggest that in a diverse study population, most participants were able to rapidly, accurately, and independently complete the application. These adaptations may offer an avenue to improve referral for LS-related genetics services.

Based on feedback from patient advisors, we included graphic and descriptive literacy aids to improve understanding and clarify difficult concepts. We also simplified the PREMM_5_™ application to allow collection of relevant cancer family health history on both sides of the family and to ask about individual relatives or groups of relatives. Our preliminary results show that in our diverse population of study participants, most appear to be able to independently complete an interactive application collecting family health history pertaining to LS risk. Participants were also able to complete the application quickly, in an average of less than five minutes. Together, these results suggest that these adaptations were successful in terms of ease of completion. Given that a major barrier in health care is the identification of individuals at risk of hereditary cancer syndromes, a tool that can be quickly and easily completed by the patient could reduce this barrier to genetics services referral, especially in the primary care setting [11–13]. Systematically providing risk assessment to all patients in primary care may help reduce the known disparities in access to referral for hereditary cancer genetic evaluation in historically underserved populations [18–22]. However, it is important to note that of the participants completing the risk assessment but requiring assistance from a study staff member (5% of the study population), almost all (96%) met the study recruitment definition of underserved and 79% of Spanish speakers utilized assistance. As such, it would be important for care systems to provide some bilingual staffing for assistance in completing such applications, in order for the tool to be most equitably deployed. Such staff would not need to be highly trained.

It is recommended that patient-facing family health history tools are evaluated for their analytic validity (how well it measures the family history that it intends to collect in comparison to a gold standard family health history collection) and time to completion [37–40]. Yet, reviews of existing family health history tools show that few studies assess these components [37–39, 44–46]. A recent review of predominantly (78%) self-administered cancer family health history tools found that researchers reported time to completion for less than half (42%) of the 62 tools reviewed [37]. Of tools with time-to-completion reported, 77% took less than 30 min to complete, with the remaining tools taking between 33 min to 120 h (no data was provided on interruptions). Thus, in comparison to the literature, PREMM_5_™ performs well in terms of time-to-completion.

While some studies have reported analytic validity of family health history tools, the methodologies vary [37]. Individuals with a concerning family health history may be missed by the risk assessment due to patient misunderstanding or misreport (false negatives)or individuals may over-report cancer history and be incorrectly classified as high risk (false positives). As such, we have utilized this as the primary metric to assess analytic validity. In our study, genetic counselors collected family health history from all patients who had genetic testing, regardless of whether they qualified via PREMM_5_™, B-RST ™ 3.0, or on the basis of limited knowledge of family history/structure. Of the individuals who qualified via means other than PREMM_5_™, our genetic counselors did not identify any additional patients with a clinically significant PREMM_5_™ score, indicating that the PREMM_5_™ application is sensitive. Thus, if this tool were implemented broadly, it may help to address the barrier of low identification of at-risk individuals.

Looking more granularly at PREMM_5_™ risk score differences between the participant-reported and genetic counselor-collected family health history, when excluding two extreme outliers, there appears to be modest agreement between the two measures (Fig. 4). This suggests that the patient-facing tool may approximate genetic counselor-collected risk assessments in many cases, but we observed a few cases of large disagreement that warrant further investigation. This investigation may allow us to isolate the source of disagreement and offer avenues to improve the tool. Additionally, we observed a trend toward overreport of family history on the patient-facing tool. It will be important to understand reasons for misreport and overreport on the patient-facing application to allow further improvement of the application, especially in terms of addressing whether the tool is equitable in diverse populations.

### Limitations

Because recruitment occurs at KPNW primarily via email and text message with a link to the CHARM webpage, we likely oversampled participants who have internet access. The initial sample is also biased toward English-speaking participants, as the Spanish application was implemented six months after the English application, and female participants outnumber male participants, which is common in genetic studies [47, 48]. More data are needed to evaluate the success of the application in individuals who choose to complete the application in Spanish, since only 14 individuals had completed it at the time of this analysis, many utilized assistance, and none had yet completed genetic counseling. Additionally, the DH population was initially enriched for higher-risk individuals, which may impact the validity analyses. Thus, participants included in our initial sample may not be representative of historically underserved populations nor the study population once the study is completed, so a full validity assessment will be required at study end. Further analyses will be required with a greater number of patients to discern possible patterns of misreport. A detailed description of recruitment results and response rates for the CHARM study is planned at study completion.

The PAC feedback regarding the risk assessment consent was greatly modified by the primary site IRB [32]. Importantly, IRB input led to greatly increased literacy level of the consent for risk assessment. This is in line with the reported experience of studies in the Electronic Medical Records and Genomics (eMERGE) network, which also reported difficulty balancing providing detailed information about genomic studies as required by site IRBs, and the need for readability [49]. Future studies should take into account potential limitations of stakeholder work in the light of IRB-required elements of consent and develop processes with site IRBs to continue to improve the navigation of the important intersection of enhancing research participant diversity and protecting the interests of vulnerable populations.

While we have examined the ability of participant self-report on the PREMM_5_™ patient-facing application to appropriately risk-stratify patients in comparison to a genetic counselor, this examination is biased toward participants who qualified for CHARM via one of three risk assessments and elected genetic testing. Thus, it is probable that the true sensitivity is lower than in this population; that is, there are additional false negatives in the population of individuals who did not qualify for genetic testing on any risk assessment, or who qualified but did not consent to genetic testing. Further, to make quantitative comparisons of PREMM_5_™ risk scores, we had to approximate participants without an evaluable PREMM_5_™ family health history input on the patient application or with no LS history as determined by the genetic counselor as having a PREMM_5_™ risk score of 0, and genetic counselors utilized the age at result disclosure in the risk calculation, which in some cases may have been one year older, resulting in a slightly lower PREMM_5_™ risk score.

Finally, family history data during genetic counseling should ideally be verified with participant and/or family member’s medical records, where possible [37–40]. Due to study limitations, this additional level of confirmation was not obtained, so the genetic counselor-recorded family history may recapitulate errors, such as misremembered ages, which would have greater chance of remedy in the traditional clinical model [38, 39].

## Conclusions and Future Directions

Our preliminary data indicate that, in the diverse CHARM study population, most participants are able to quickly complete the PREMM_5_™ patient-facing application. Early data suggest the PREMM_5_™ application can appropriately risk-stratify most patients compared to genetic counselor-collected family health history. However, there are a few patients who have relatively large PREMM_5_™ score differences, as well as patients who are inappropriately categorized. Participants typically overestimate the frequencies of LS-related cancers in their family on the patient-facing application. We plan to conduct a validity analysis with all patients at study end, to analyze patterns in misreport, and to examine the contribution of participant characteristics such as education, English fluency, and numeracy to the time participants spent on the application and to the accuracy of their family health history input. Future work will also include qualitative interviews with participants with variations in misreport. These evaluations will be used to refine the PREMM_5_™ patient application to create an equitable assessment with higher specificity.

## Supplementary Information

Below is the link to the electronic supplementary material.

Supplementary Figure 1. Sample images from the Spanish patient-facing PREMM_5_^TM^ application. (A) Sample screen inquiring about cancer history in individual relatives or small groups of relatives. (B) Sample screen inquiring about cancer history in the mother, which appears if the participant selects that the mother had cancer. (C) Sample literacy aid pop-up window depicting the family tree graphic, which appears if the patient selects the modal link titled “¿Quiénes son mis parientes directos?” on the screen in A. The pop-up literacy aid for sebaceous gland skin tumors that appears when the patient selects the modal link in B is depicted in Supplementary Figure 2B (PNG 760 KB)

Supplementary Figure 2. Pop-up literacy aid describing sebaceous gland skin tumors in English (A) and Spanish (B). The PAC provided feedback that clarification was needed around this cancer concept. The study team responded by creating a pop-up literacy aid, the final version of which is displayed (PNG 299 KB)

Supplementary Figure 3. Explanation of Lynch syndrome in English (A) and Spanish (B). The PAC provided feedback that clarification was needed around what the application results told them. They advised that information related to Lynch syndrome was appropriate to include in the risk assessment results for participants exposed to the PREMM_5_^TM^ application. The final version from the risk assessment results report is displayed (PNG 666 KB)

Supplementary Figure 4. Flowchart of participant movement through risk assessment stages. Participant movement through risk assessment stages for the total number of unique individuals included in this manuscript, which includes 500 included in the time analysis who were exposed to PREMM_5_^TM^-specific questions, 1 participant who was excluded from the time analysis on the basis of data recording errors but who was exposed to PREMM_5_^TM^-specific questions and included in the validity analysis, and 16 individuals who were not exposed to PREMM_5_^TM^-specific questions but were included in the validity analysis to assess for false negatives (PNG 181 KB)

Supplementary Table 1 (DOCX 69 KB)
