# Supplementary figures and images for: Adaptation and early implementation of the PREdiction model for gene mutations (PREMM5™) for lynch syndrome risk assessment in a diverse population
Source: Fam Cancer. 2021 Mar 23;21(2):167–80. doi: 10.1007/s10689-021-00243-3 (PMC8458476; doi:10.1007/s10689-021-00243-3)

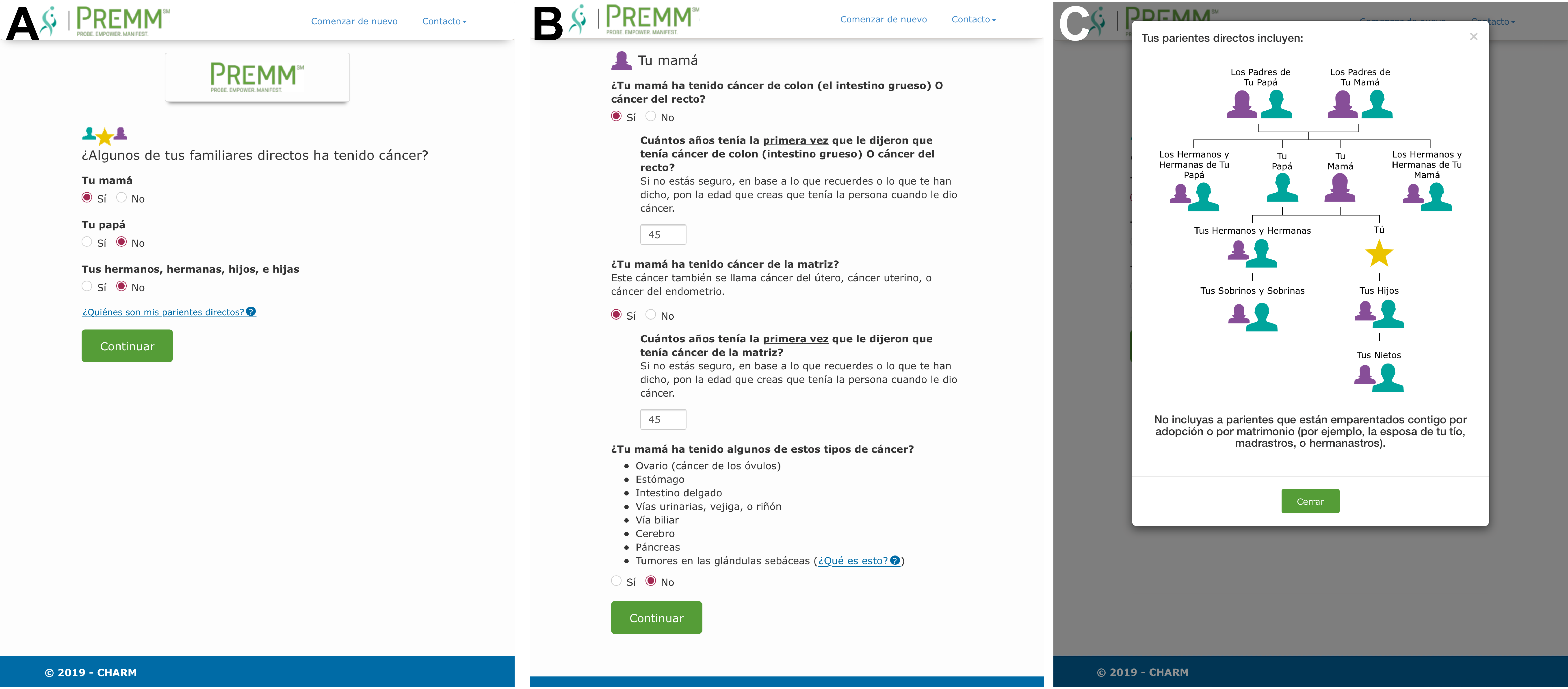

Supplement: Supplementary file 1 — Supplementary Figure 1. Sample images from the Spanish patient-facing PREMM5TM application. (A) Sample screen inquiring about cancer history in individual relatives or small groups of relatives. (B) Sample screen inquiring about cancer history in the mother, which appears if the participant selects that the mother had cancer. (C) Sample literacy aid pop-up window depicting the family tree graphic, which appears if the patient selects the modal link titled “¿Quiénes son mis parientes directos?” on the screen in A. The pop-up literacy aid for sebaceous gland skin tumors that appears when the patient selects the modal link in B is depicted in Supplementary Figure 2B (PNG 760 KB) [file 10689_2021_243_MOESM1_ESM.png]

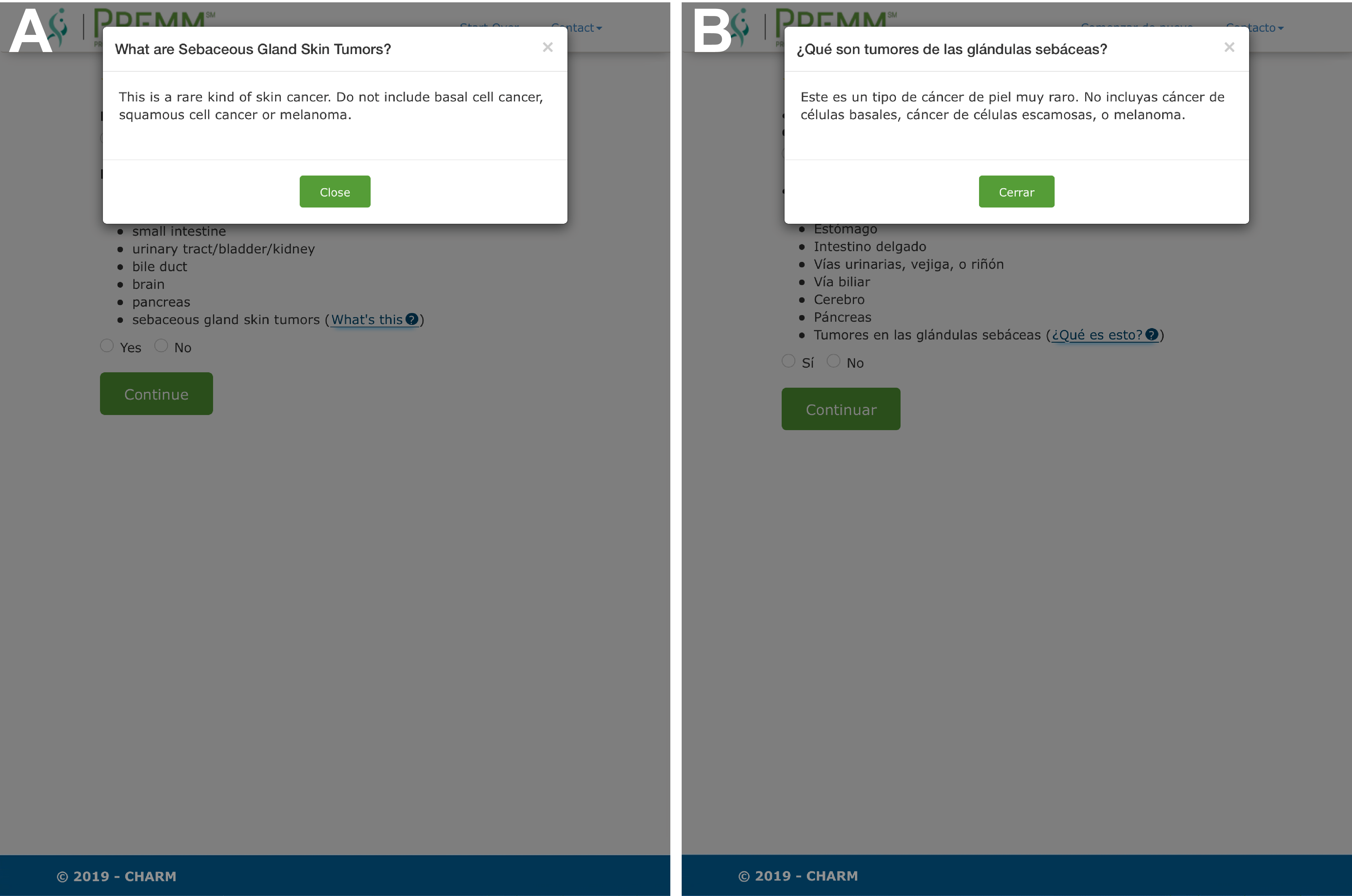

Supplement: Supplementary file 2 — Supplementary Figure 2. Pop-up literacy aid describing sebaceous gland skin tumors in English (A) and Spanish (B). The PAC provided feedback that clarification was needed around this cancer concept. The study team responded by creating a pop-up literacy aid, the final version of which is displayed (PNG 299 KB) [file 10689_2021_243_MOESM2_ESM.png]

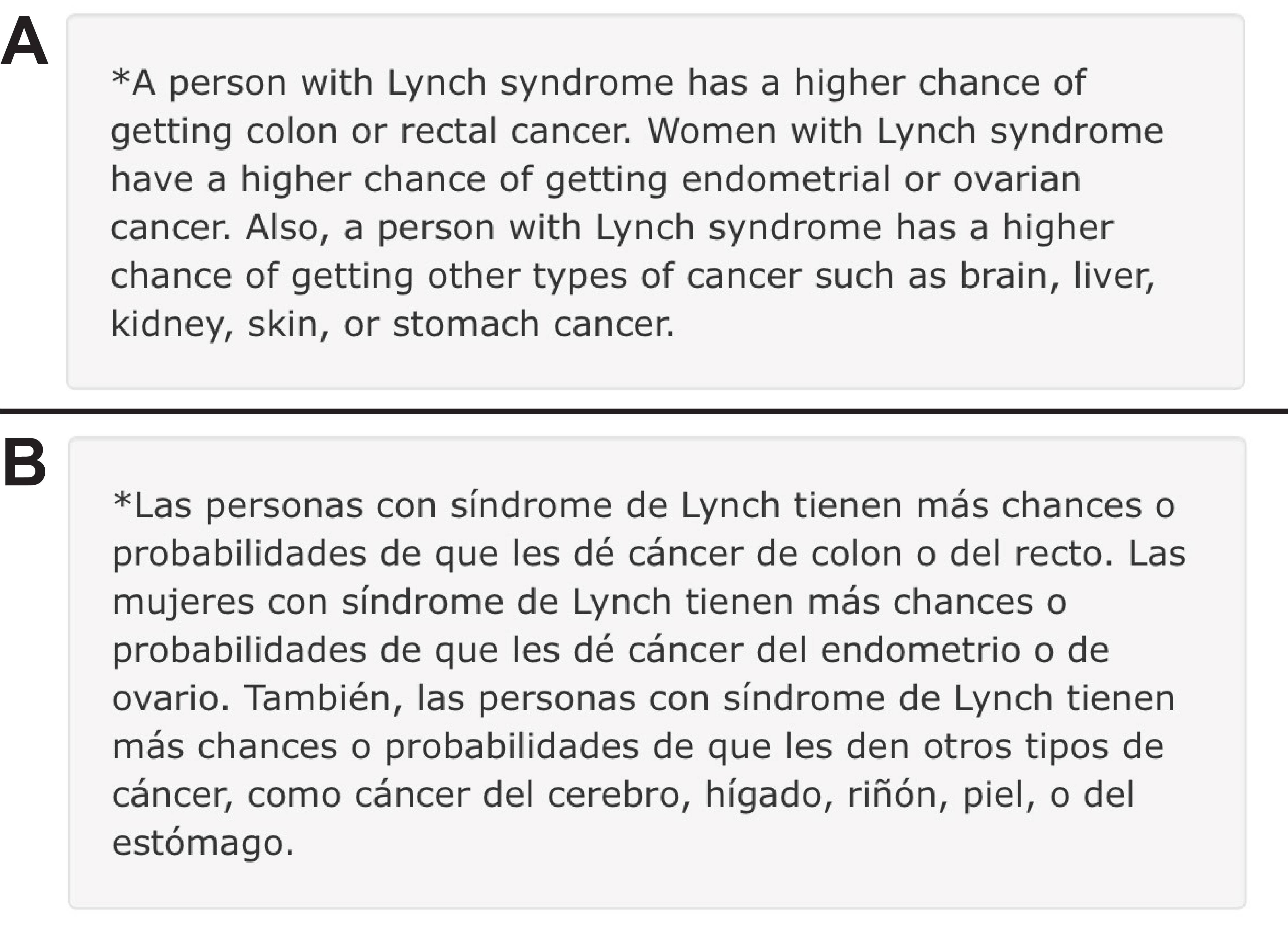

Supplement: Supplementary file 3 — Supplementary Figure 3. Explanation of Lynch syndrome in English (A) and Spanish (B). The PAC provided feedback that clarification was needed around what the application results told them. They advised that information related to Lynch syndrome was appropriate to include in the risk assessment results for participants exposed to the PREMM5TM application. The final version from the risk assessment results report is displayed (PNG 666 KB) [file 10689_2021_243_MOESM3_ESM.png]

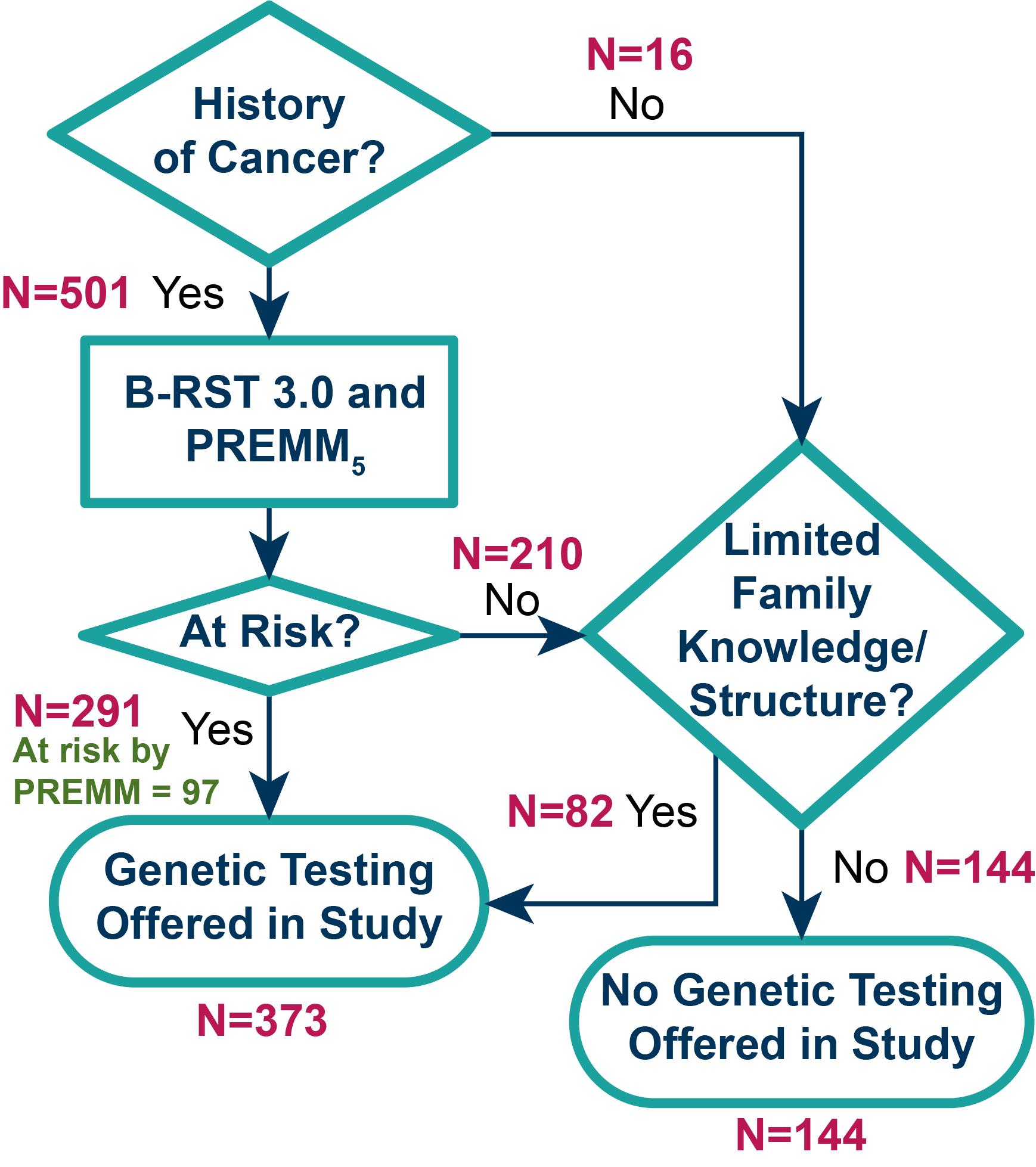

Supplement: Supplementary file 4 — Supplementary Figure 4. Flowchart of participant movement through risk assessment stages. Participant movement through risk assessment stages for the total number of unique individuals included in this manuscript, which includes 500 included in the time analysis who were exposed to PREMM5TM-specific questions, 1 participant who was excluded from the time analysis on the basis of data recording errors but who was exposed to PREMM5TM-specific questions and included in the validity analysis, and 16 individuals who were not exposed to PREMM5TM-specific questions but were included in the validity analysis to assess for false negatives (PNG 181 KB) [file 10689_2021_243_MOESM4_ESM.png]
