# Supplementary material for: Adaptation and early implementation of the PREdiction model for gene mutations (PREMM5™) for lynch syndrome risk assessment in a diverse population
Source: Fam Cancer. 2021 Mar 23;21(2):167–80. doi: 10.1007/s10689-021-00243-3 (PMC8458476; doi:10.1007/s10689-021-00243-3)
Supplement: Supplementary file 5 — Supplementary Table 1 (DOCX 69 KB) [file 10689_2021_243_MOESM5_ESM.docx]

**SUPPLEMENTAL TABLE**

**Supplementary Table 1. Comparison of mutually exclusive populations of individuals exposed to PREMM_5_^TM^-specific questions and included in the analysis of time and incompletion, but not yet receiving genetic counseling (N = 393), to the individuals included in the validity analysis (N = 124).**

|  | **Individuals taking the RAT but not receiving GC at time of analysis**  **(N = 393)** | **Individuals included in validity analysis**  **(N=124)** |  |  |
| --- | --- | --- | --- | --- |
| **Variable** | **N (%)*** | **N (%)** | **Effect size (Odds Ratio) [95% CI]** | **p-value** |
| Sex assigned at birth  *Male*  *Female* | 76 (19.3%)  317 (80.7%) | 23 (18.6%)  101 (81.5%) | 1.05 [0.63,1.77] | .85 |
| Site  *KP*  *DH* | 301 (76.6%)  92 (23.4%) | 99 (79.8%)  25 (20.2%) | 0.83 [0.50,1.36] | .45 |
| Application Language Selection  *English*  *Spanish* | 379 (96.4%)  14 (3.6%) | 124 (100%)  0 (0%) |  | .028 |
| Education^†^  *Some high school or less*  *High school graduate*  *Some post-high school training*  *Associate or vocational degree*  *Bachelor's degree*  *Graduate or professional degree* | 14 (9.5%)  18 (12.2%)  41 (27.9%)  17 (11.6%)  34 (23.1%)  23 (15.7%) | 4 (3.8%)  17 (16.0%)  28 (26.4%)  17 (16.0%)  22 (20.8%)  18 (17.0% | Ref  0.30 [0.09,0.98]  0.42 [0.13,1.30]  0.29 [0.09,0.90]  0.44 [0.14,1.37  0.37 [0.12,1.14] | .07  Ref |
| English Fluency^‡^  *Native English-speaker*  *Non-native** | 118 (78.7%)  32 (21.3%) | 91 (86.7%)  14 (13.3%) | 0.57 [0.29,1.13] | .10 |
| Age at time of risk assessment (years)  @35.3 years  @40.3 years | 68.5%  69.8% | 31.5%  30.2% | 0.96 [0.85,1.08] | .49 |
| SNS-3 Score^§^  @12.9  @13.9 | 27.9%  30.4% | 72.1%  69.6% | 0.97 [0.91,1.03] | .26 |

*Significant at alpha level .05

^†^Percentages are given out of the number of individuals with available data for this question on the survey (N = 147 for the reference population and N = 106 for the validity analysis group). As the overall p value was not significant at alpha .05, individual level p values were not reported.

^‡^ Percentages are given out of the number of individuals with available data for this question on the survey (N = 150 for the reference population and N = 105 for the validity analysis group); not all individuals in the reference population were eligible for the survey, which was only presented to participants eligible for genetic testing through the study. Native speakers were considered those who answered the survey in English and answered that they did not speak a language besides English, or those who answered that they spoke a language besides English but chose English as their native language. Individuals taking the survey or risk assessment in Spanish, and those took the baseline in English and who selected that they were a non-native English speaker were included as non-native English speakers. Individuals taking the survey in English but not answering questions about English fluency were treated as missing.

^§^Assessed only for individuals completing this portion of the baseline survey, which was available only to participants with a genetic testing-eligible risk assessment outcome.
